# Progress in State Medicine

**Published:** 1901-05-04

**Authors:** 


### Progress in State Medicine.

**Tuberculosis.**—Swithinbank,[1] discussing dust in relation to tuberculosis, states that by far the greater proportion of infection is due to small particles of the desiccated phlegm of consumptives carried to and fro with the dust by air currents. This danger is ever present and real until consumptives realise that they are a standing danger to the community by their promiscuous spitting in streets and other public places. The bodies examined at the Morgue, death being from violence or causes entirely apart from tuberculosis, show that half of those who have passed thirty years of age had cicatrised tubercles in the lungs. Marked decrease in the habit of spitting will be effected when the danger is instilled into the public mind. Common sense and education of the young will do the rest. Cornet, of Berlin, confined at different levels forty-eight guinea-pigs in a room the carpet of which had been smeared with the phlegm of a patient suffering from advanced tuberculosis of the lungs. The phlegm was allowed to dry and the carpet then vigorously brushed. Of the forty-eight guinea-pigs introduced forty-six became infected with tuberculosis. In France notices against spitting are being affixed in public places, railway stations, streets, and in places of entertainment. The railway companies are seeking powers, through their by-laws, to eject from their premises people guilty of the habit. Wet swabbing and the use of wet cloths is to supersede the brush on platforms and in carriages. Spittoons in prominent places are already provided at some of the stations. Cushions, carpets, and hangings are to be properly beaten in enclosed spaces. Blankets and rugs used by passengers are to be disinfected after each journey.

Girdansky[2] refers to the fact that the bacilli do not enter the lungs of healthy persons directly from the lungs of the sick, the breath of tuberculous patients not being infective. The danger is from the dried bacilli floating about attached to minute particles of dust. He attributes the production of this dust not so much to every-day traffic aided by the action of the wind, as to the over-energetic use of the housewife's broom. The larger dirt is removed at the expense of the air being filled with infected dust. He maintains that sweeping breaks up all dried sputum and other excretions, thus setting free the micro-organisms in them, transmuting them from a low innocuous latent state to a more virulent condition. These bacilli are kept floating in the air from the repeated use of the broom. Therefore the broom must be abolished; the carpets being breeding grounds for vegetable parasites should be replaced by a healthier substitute. Cleansing of floors and streets should be accomplished with mops, showers and sprinkling wagons. All floors and floor coverings of the home and street ought to be so constructed as to allow of this method of cleansing.

Rube[3] draws attention to the sanatoria provided for the working classes in Germany. Statistics of the Imperial Board of Health showed that 343 people died in 1893, between the ages of 15 and 60, out of every 1,000, of tuberculosis; and the statistics of the Imperial Board of Insurance which dealt with 158,000 cases from 1890 to 1895 show that out of every 1,000 sick cases recorded between the ages of 20 to 30, tuberculosis was responsible for 542. By law every workman and workwoman from the age of 16, who earns not more than £100 in the year, is compelled to insure with the State. About 35 millions out of a population of 53 millions derive support from this law. The State insurance institutes, of which there are 31, are allowed to build sanatoria for the treatment of the insured. Forty-five sanatoria now exist, and it will soon be possible to treat yearly 20,000 patients in Germany, each for about three months. Most of the patients return home cured; the remainder having been trained in hygienic methods are not so likely to be injurious to others. Figures show that 65·7 per cent. of 2,259 patients who left the different sanatoria were able to take up their former trade, 6·5 per cent. had to find more suitable occupation, 12·8 per cent. could partly follow their former vocation, whilst only 15 per cent. were sent home unfit for work. Early diagnosis is essential for successful results. The State Insurance Institute, Hanover, has treated up to the present 5,000 of their insured patients, who are carefully watched as to their state of health and earning capacity for five years after leaving the sanatoria. The figures for 1892–3 prove in satisfactory manner that the expenses incurred in these years throughout the treatment of the insured have been amply covered by the saving in pensions.

At a meeting[4] held in support of providing a sanatorium for the counties of Gloucester, Somerset and Wilts, for the open-air treatment of poor, but not rate-aided consumptives, Crichton-Browne stated his belief that the sanatorium treatment had come to stay. It would accomplish good results if kept well under medical control and free from commercial entanglements. It seemed clear that if cases in the first stage of consumption were thoroughly treated in good time, 50 per cent. of radical cures would be effected, and a marked improvement in another 25 per cent. The scheme for carrying on the sanatorium when built, was that each of the three counties should provide from £300 to £400 per annum; the rest they hoped to get by persuading the poor to form themselves into groups of 300 in the different towns and districts, each member to pay a penny a week, in return for which each group would have a claim upon the sanatorium to send three or four patients to be treated.

Parker[5] thinks that little headway will be made in this country against tuberculosis until there is uniform action. The best preventive measures are notification of phthisis and free disinfection of infected dwellings vacated by consumptives. These would facilitate adequate sanatoria being provided for the curable cases, and isolation homes for the incurable. Attention would be more directly called to overcrowded, badly-ventilated dwellings, and improved subsoil drainage would be provided for the damp districts. All rooms occupied by consumptives should be frequently cleansed. Patients not under the charge of a medical man should be dealt with by the medical officer of health. The objection to notification as being a breach of medical confidence can be overcome by securing the permission of the patient. If he refuses and continues a dangerous source of infection, notification should take place without his consent. The objection to medical officers of health interfering with private patients can be avoided by such officials working through the private practitioner, provided the latter has real supervision of the case. In Brighton, Manchester, and Sheffield, where the usual notification fees are paid, very satisfactory results have been obtained. In other towns where the fee is only a shilling the system is a failure. In Norwich, for example, there were eleven notifications in the first three or four months, none at all during the following six months. Very few cases have been notified in Liverpool, Cardiff and Blackpool where no fees are paid. So complete has been the failure in some of these towns that adequate fees are about to be proposed. The mere notification by registrars of deaths due to phthisis, even though accompanied by the disinfection of the dwellings concerned, as practised in some towns, seems an inadequate measure.

Kerr [6] remarks that the actual disease, phthisis, is rarely seen in a Board School, an affected child ceasing to attend at an early age; but with children in institutions it may be quite different. Cases of suspected phthisis are seen about as rarely as noticeable lupus; cases of tubercular bone disease are altogether at least five times as common as the lung disease. Statistics show that in the case of phthisis there appears an increased death rate, associated with increased school attendance. Many cases are reported as consumptive which are merely anæmic or neurotic. Others diagnosed as chronic bronchitis are undoubtedly phthisis. The Bradford School Board has adopted the rule that in doubtful phthisical cases, if the child coughs or spits it shall be excluded from the school until it can with safety attend regularly, or have its name removed from the register, meanwhile having its attendance marked as if infectious. In 1,000 carefully examined cases, recorded at the time when attendance officials and the parents differed as to the advisability of school attendance, 3 per cent. were phthisical, 4 per cent. had obvious tuberculous disease, chiefly bone, 3 per cent. chronic bronchitis, 6 per cent. rheumatic class of diseases, 3 per cent. photophobia or keratitis, 4 per cent. chronic otorrhœa, 7 per cent. obviously adenoids, and 10 per cent. vague sickness with debility, whilst 40 per cent. were quite fit for school. Phthisis as a direct evil at school ages may be regarded as almost negligible. Anything that debilitates, however, prepares for phthisis at a later stage. Town children require more opportunity for games involving heavy muscular exercise than those in the country. Defective ventilation, which is the rule in schools, is the most important debilitating factor, the younger children being affected most. Natural ventilation cannot be admitted as sufficient for schoolrooms. Not less than 1,000 cubic feet per head per hour is needed, and this requires artificial currents produced by fans for large schools—the smaller rooms can be managed with heated flues. School management from the sanitary point of view is a subject quite neglected in the education of teachers; it should be taught in the training colleges and required in the scholarship and certificate examinations. A pamphlet dealing with personal cleanliness, proper clothing, recreation, sleep, and sufficient fresh air is distributed in thousands throughout the Bradford schools.

[1] Tuberculosis, Jan. 1901. [2] Med. Jour. N.Y., Sept. 9, 1899. [3] Tuberculosis, Jan. 1901. [4] Brit. Med. Jour., Mar. 23, 1901. [5] Brit. Med. Jour., Oct. 13, 1900. [6] Tuberculosis, July, 1900.

